# Prediction of future input explains lateral connectivity in primary visual cortex

**DOI:** 10.1016/j.cub.2024.11.073

**Published:** 2025-01-10

**Authors:** Sebastian Klavinskis-Whiting, Emil Fristed, Yosef Singer, M. Florencia Iacaruso, Andrew J. King, Nicol S. Harper

**Affiliations:** 1 Department of Physiology, Anatomy and Genetics, University of Oxford, Parks Road, Oxford OX1 3PT, UK

## Abstract

Neurons in primary visual cortex (V1) show a remarkable functional specificity in their pre- and postsynaptic partners. Recent work has revealed a variety of wiring biases describing how the short- and long-range connections of V1 neurons relate to their tuning properties. However, it is less clear whether these connectivity rules are based on some underlying principle of cortical organization. Here, we show that the functional specificity of V1 connections emerges naturally in a recurrent neural network optimized to predict upcoming sensory inputs for natural visual stimuli. This temporal prediction model reproduces the complex relationships between the connectivity of V1 neurons and their orientation and direction preferences, the tendency of highly connected neurons to respond more similarly to natural movies, and differences in the functional connectivity of excitatory and inhibitory V1 populations. Together, these findings provide a principled explanation for the functional and anatomical properties of early sensory cortex.

## Graphical abstract

**Graphical abstract:**
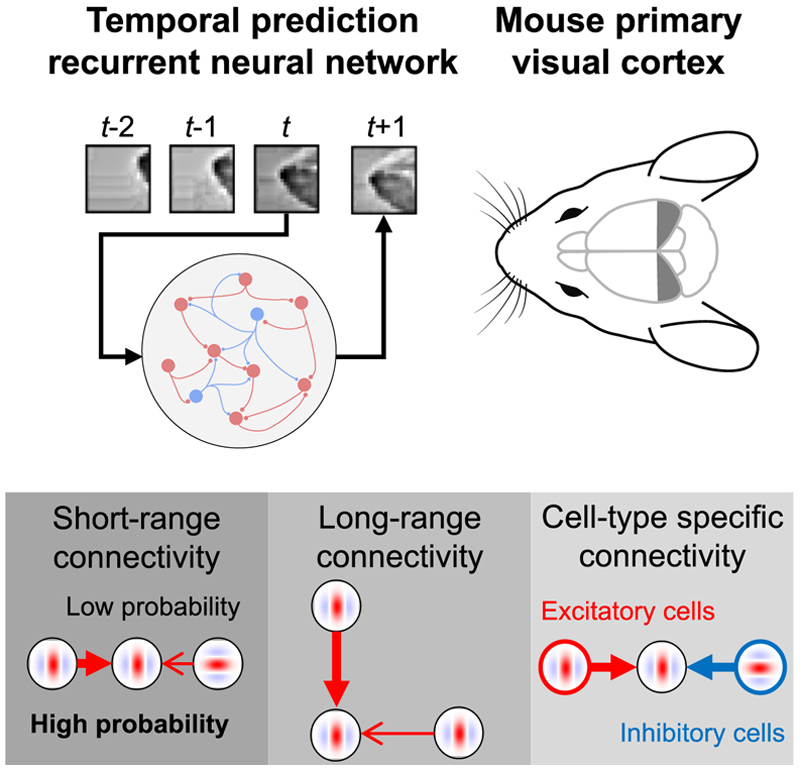

## Introduction

An increasing number of studies—mostly focusing on mouse primary visual cortex (V1)—have begun to uncover the underlying rules specifying how cortical neurons connect.^1–4^ Some findings, such as the tendency of V1 neurons to synapse with other neurons that show similar orientation selectivity, follow a simple like-for-like pattern.^2–4^ By contrast, other results, such as the spatial organization of synaptic inputs to orientation- and directiontuned visual neurons, appear more complex and less amenable to a unifying theoretical explanation.^1,5^ An outstanding question, then, is how to understand these putative connectivity rules and whether they can be explained by a single general principle.

By taking a normative approach, we can ask whether the patterns of structure and function observed at the level of individual neurons are optimized for achieving a particular goal that is important behaviorally or from an evolutionary perspective. One such promising normative framework is that of temporal prediction, which posits that sensory systems are optimized to represent those features in the recent past that are predictive of the immediate future sensory inputs.^6,7^ Why might temporal prediction serve as a useful objective for an organism’s sensory systems? First, if sensory systems construct a model of the world, then a good model should predict future inputs well.^8,9^ Second, predictive features are valuable for guiding actions and for compensating for neural conduction and processing delays,^10^ enabling, for example, a cat to catch a bird in flight. Third, extracting predictive features reduces the vast amount of information the brain needs to manage. Finally, temporal prediction requires no explicit teaching signal beyond the sensory input itself, making it inherently more biologically plausible as an unsupervised principle than other supervised counterparts.^11,12^

When optimized for temporal prediction, feedforward networks have been shown to capture many of the receptive field characteristics and response properties of V1 neurons, as well as motion processing along the visual pathway.^6,7^ Nevertheless, existing feedforward temporal prediction models neglect the role of recurrency, which experimental and theoretical studies have implicated in a range of key brain functions.^13–15^

Here, we show that a recurrent network optimized for temporal prediction on dynamic natural visual scenes can capture many motifs of local connectivity in visual cortex. Furthermore, when we compared network models optimized for different normative objectives, temporal prediction stood out in its capacity to explain these connectivity motifs. Hence, the relationship between the connectivity patterns of V1 neurons and their response characteristics appears to be optimized to support the predictive processing of dynamic stimuli.

## Results

### Model response properties

**Figure 1:**
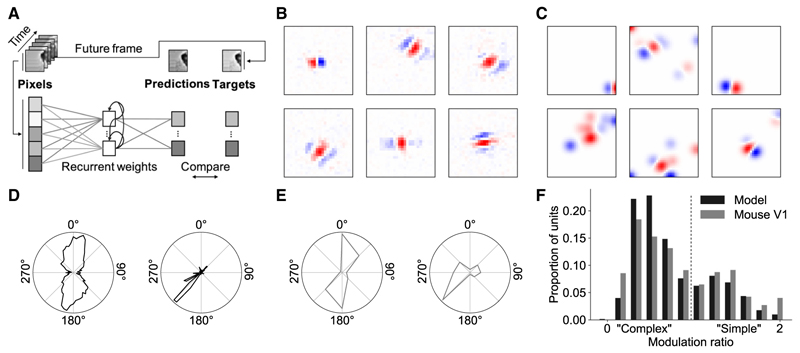
The recurrent temporal prediction model captures basic tuning properties of V1 neurons (A) Schematic of the recurrent temporal model. 2,332 hidden units (90%) were excitatory with non-negative outgoing recurrent weights, and the remaining 260 hidden units (10%) were inhibitory with non-positive outgoing recurrent weights. (B) Response-weighted-average receptive field estimates of model units (units in pixels). (C) Mouse V1 receptive fields from publicly available recordings,^17^ pre-processed for visualization by thresholding and smoothing with a Gaussian filter (units in pixels). (D) Example tuning curves for orientation (left) and direction (right) tuned model units. Response is the normalized hidden unit activity as a function of the stimulus value. (E) Example tuning curves for orientation- and direction-tuned cells in V1.^17^ Response is the normalized firing rate as a function of the stimulus value. (F) Distribution of modulation values across model and pooled excitatory and inhibitory mouse V1 units^18^; typically a modulation ratio <1 is taken as a complex cell and >1 as a simple cell. See also Figures S1 and S2.

We trained a recurrent network model to predict the upcoming visual input (40 ms ahead) based on the recent stimulus history (Figure 1A). The model was trained on a diverse dataset of movies of natural scenes, including wildlife and panning over natural environments, which were bandpass filtered to approximate the retina.^16^ After training, we first compared model hidden unit response properties with those of neurons in mouse V1.

We estimated the model units’ receptive fields by means of the response-weighted average (Figure 1B). Like simple cells in V1 (Figure 1C), model units often had well-defined receptive fields with a Gabor-like structure consisting of oriented excitatory and inhibitory subfields. To probe the tuning properties of the model units, we recorded the model’s responses to oriented full-field drifting gratings. Model units were generally orientation tuned (24%) or direction tuned (57%; Figures 1D and 1E), with a similar distribution of orientation selectivity indices (OSI) and direction selectivity indices (DSI) as found in mouse V1 (Figure S1). As in visual cortex, model units varied in their phase responsiveness, characterized by their modulation ratio (Figure 1F). Highly modulated units were classed as simple-cell like, while units that displayed little or no phase modulation were classed as complex-cell like.^19,20^ At the population level, there was a bimodal distribution similar to that found in mouse V1,^18,21^ indicating the existence of distinct populations of simple-cell-like and complex-cell-like model units (Figure 1F).

Some neurons in V1 are thought to encode prediction errors.^22,23^ While the model hidden units do not explicitly represent prediction errors by default, they could emerge spontaneously during the inference process in response to “unexpected” stimuli that violate a recurring pattern (Figure S2A). We found a small number of units that responded selectively to these unexpected stimuli (omitted stimuli: 0.2% of units; deviant stimuli: 5.7% of units), while a slightly larger number of model units showed mixed sensitivity and responded to both standard and unexpected stimuli (omitted stimuli: 0.5% of units; deviant stimuli: 9% of units). Prediction-error responses have been reported in some neurons in mouse V1^23–25^ and to varying degrees in other primary sensory areas.^26–30^ Moreover, this mixed sensitivity aligns with the biology where neurons rarely exist in a strictly error-coding capacity.^25,31^

### Short- and long-range functional connectivity

**Figure 2:**
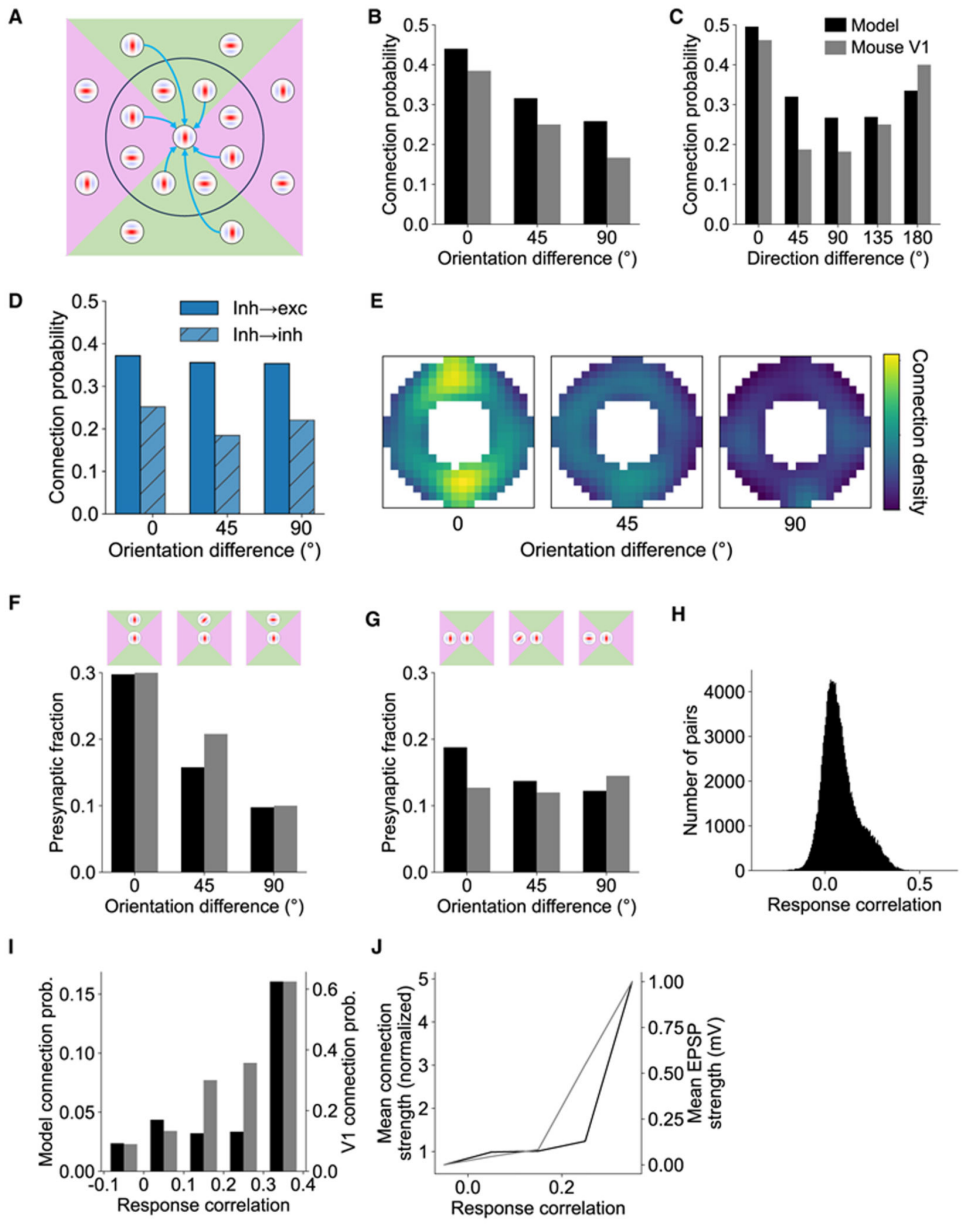
The model captures short- and long-range functional connectivity in V1 (A) Schematic of short- and long-range connectivity in V1. Short-range connections are more prevalent for similarly tuned V1 neurons, whereas long-range connection probability is greater for V1 neurons with similar orientation-tuning when their receptive fields are located in co-axial space. (B and C) Short-range connections are more prevalent when excitatory model units have similar orientation tuning (B) and for direction-tuned units that have similar or opposite preferred directions of motion (C), as is also the case in V1.^3^ (D) As in (B), but for inhibitory-to-inhibitory and inhibitory-to-excitatory connections in the model. (E–G) In both the model and V1,^1^ long-range connection probability is higher for presynaptic model units with similar orientation preferences when their receptive fields are located in co-axial (F) than in co-orthogonal (G) locations relative to the receptive field of the postsynaptic unit. For (B), (C), (F), and (G), data are binned using the same convention as in Ko et al.^3^ with orientation bins of 0° –22.5°, 22.5° –67.5°, and 67.5° –90° and motion direction bins of 0° –22.5°, 22.5° –67.5°, etc. Heatmap (E) shows the normalized connection probability over visual space across differences in orientation tuning for model units. Heatmap is smoothed for display purposes with a Gaussian filter (standard deviation, σ = 2 pixels). (H) Histogram of the response correlation distribution across pairs of connected model units for natural stimuli. The distribution is right skewed, indicating that a minority of model units have highly correlated responses. (I and J) As for mouse V1,^2^ response correlation for model units co-varies with the connection probability (I) as well as the input connection strength (J). These results were abolished after randomly shuffling the recurrent weights between the units when measuring connectivity, resulting in uniform distributions (Figure S4). Accordingly, the model connectivity biases cannot be explained by the underlying distribution of orientation- and direction-tuning preferences among model units. See also Figures S2–S4.

Short-range functional connectivity between excitatory units in the model resembled that of mouse V1 neurons (Figure 2A).^1,3^ Model units were more likely to synapse with other units with the same orientation preference (Figure 2B), with the connection probability monotonically decreasing as the difference in orientation preference increased (*p* < 0.0001, Cochran-Armitage). Likewise, connectivity between direction-tuned excitatory model units resembled that of mouse V1 neurons.^3^ Model units were most likely to connect when tuned to either the same or opposite direction of motion (both *p* < 0.0001, Cochran-Armitage), with connection probability decreasing as the presynaptic unit’s tuning became more orthogonal to the postsynaptic unit’s preferred direction (Figure 2C).

For inhibitory model units, co-tuning with the postsynaptic unit was much weaker (Figure 2D), as has been previously reported in V1.^5,32,33^ Neither inhibitory-to-excitatory (*p* = 0.102, Cochran-Armitage) nor inhibitory-to-inhibitory (*p* = 0.606, Cochran-Armitage) model unit connections showed a significant linear dependence of connection probability on the difference in orientation preference. For direction-tuned inhibitory-to-excitatory model units, the model predicts a weak but significant monotonic trend of increasing connection probability as the difference in preferred direction increases (Figure S3; *p* = 0.032, Cochran-Armitage), distinct from the u-shaped trend observed for excitatory model units and excitatory V1 neurons. No significant trend was found for inhibitory-to-inhibitory direction-tuned model units (*p* = 0.484, Cochran-Armitage). Finally, for orientation- and direction-tuned excitatory-to-inhibitory model units, the model predicts a similar trend as for excitatory-to-excitatory model units and V1 neurons (*p* < 0.0001, Cochran-Armitage), albeit with the minimum connection probability shifted to the 135° bin for direction-tuned model units (Figure S3).

We further investigated how connectivity differs among units with simple-cell-like versus complex-cell-like responses (Figures S3E and S3F). Overall, we found few differences between these two populations. For the short-range connectivity motifs investigated (Figures S3E and S3F), the connectivity pattern was qualitatively similar to the population distributions described above (Figures 2B and 2C), though the aggregate connection probability was higher for simple-cell-like units compared with complex-cell-like units.

Across long-range connections, the model also replicated the dependence of connectivity on neuronal orientation preference and receptive field location found in visual cortex.^1^ We measured the connection probability between pre- and postsynaptic model units as a function of their difference in preferred orientation and the presynaptic unit’s receptive field location in visual space relative to that of the postsynaptic unit (Figure 2E). As for mouse V1, model units were more likely to project to the postsynaptic unit if their receptive field aligned along the axis of the postsynaptic unit’s receptive field. To quantify this effect, we divided visual space relative to the postsynaptic unit into four quadrants. Those quadrants that aligned with the postsynaptic unit’s orientation tuning were referred to as “co-axial” space (green regions in Figure 2A), while those quadrants orthogonal to the unit’s preferred orientation were referred to as “co-orthogonal” space (pink regions in Figure 2A). As with the biology, orientation-tuned model units were more likely to synapse with other units when they had similar orientation selectivity and their receptive fields were located in co-axial visual space (Figure 2F; *p* < 0.0001, permutation test). Although a similar effect was found for co-orthogonal units (Figure 2G; *p* < 0.0001, permutation test), this relationship was much weaker. In particular, there was a significantly higher proportion of model units in the 0° orientation difference bin and a significantly lower proportion in the 90° bin for receptive fields in co-axial versus co-orthogonal space (both *p* < 0.001, permutation test).

As in V1,^2,4^ model units whose responses to natural movies were highly correlated were also more likely to be connected. In both cases, the distribution of correlation values between pairs of connected model units was skewed, with the majority of pairs of units showing a relatively low response correlation and a smaller proportion that were highly correlated (Figure 2H). A qualitatively similar relationship was seen for both model units and V1 neurons between the response correlation and both the connection probability (Figure 2I; *p* < 0.001, Cochran-Armitage) and the average strength of those connections (Figure 2J).

### Excitatory-inhibitory functional connectivity of direction-tuned units

**Figure 3:**
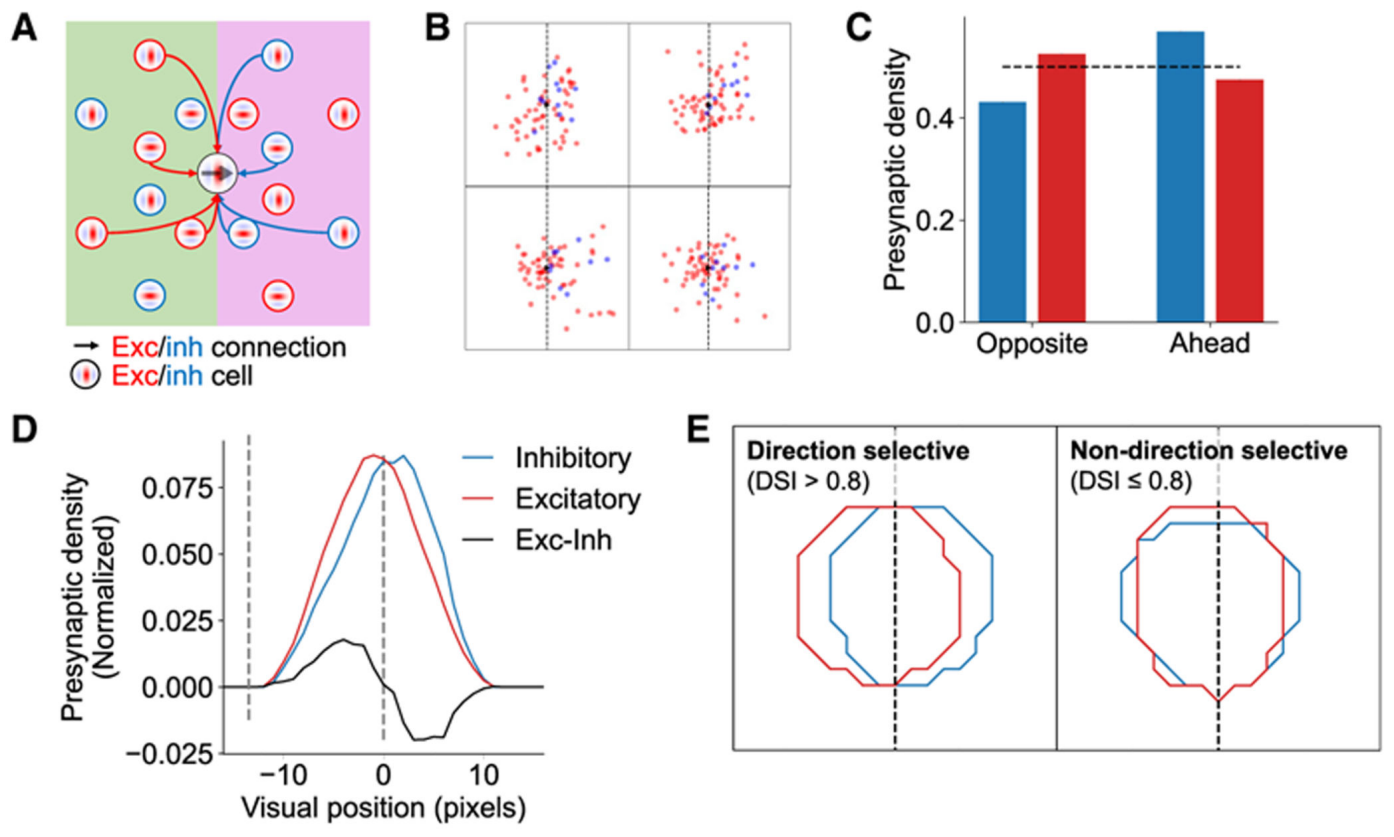
The model captures direction-dependent differences in functional connectivity between excitatory and inhibitory populations in V1 (A) Schematic of connectivity biases in excitatory and inhibitory inputs to direction-tuned cells in V1.^5^ (B) Exemplar excitatory and inhibitory presynaptic ensembles for direction-tuned excitatory model units. (C) Model unit presynaptic density for excitatory and inhibitory subpopulations in the sectors of visual space opposite to and ahead of the post-synaptic unit’s preferred direction of motion. Dashed line represents equal density (0.5). (D) Profile of model presynaptic unit location density across horizontal visual space for excitatory and inhibitory inputs. Profiles smoothed with a 5-pixel moving average. (E) Pooled location density contours over visual space across all excitatory (red) and inhibitory (blue) model units for direction- and non-direction-selective postsynaptic excitatory units. These results were abolished after randomly shuffling the recurrent weights between the units when measuring connectivity, with no difference in density across either sector of visual space for excitatory or inhibitory model units (Figure S4). See also Figure S4.

As for orientation-tuned cells, synaptic inputs to direction-tuned cells in V1^5^ are not uniformly distributed in visual space (Figure 3A). In particular, direction-tuned excitatory cells preferentially receive inputs from other excitatory cells whose receptive fields are situated in the opposite sector of visual space (i.e., behind) the postsynaptic cell’s preferred direction. By contrast, the opposite effect is observed for inhibitory cells, which preferentially synapse with excitatory cells if the location of their receptive fields is ahead of the postsynaptic cell’s preferred direction of motion. This connectivity motif provides a plausible circuit basis for direction selectivity, whereby a spatial offset combined with a conductance delay for inhibitory cells facilitates the detection of moving stimuli.^5^

In line with this evidence from V1, excitatory presynaptic ensembles in the model were more numerous in the opposite sector relative to the postsynaptic unit’s preferred motion direction (Figures 3B–3E; *t*(113) = 2.93, *p* = 0.004). Conversely, the opposite pattern was found for the inhibitory model units, whose connection probability with direction-tuned excitatory units was higher if their receptive fields were located in the sector of visual space ahead of the preferred direction of motion (Figures 3B–3E; *t*(113) = −2.80, *p* = 0.006). This effect was specific to direction-tuned excitatory model units, with no significant difference in the spatial locations of excitatory and inhibitory presynaptic ensembles synapsing with weakly direction-selective units, as defined by a DSI ≤ 0.8 (see STAR Methods; Figure 3E; *t*(113) = 225, *p* = 0.337).

### Comparing V1 response prediction and connectivity across models

**Figure 4:**
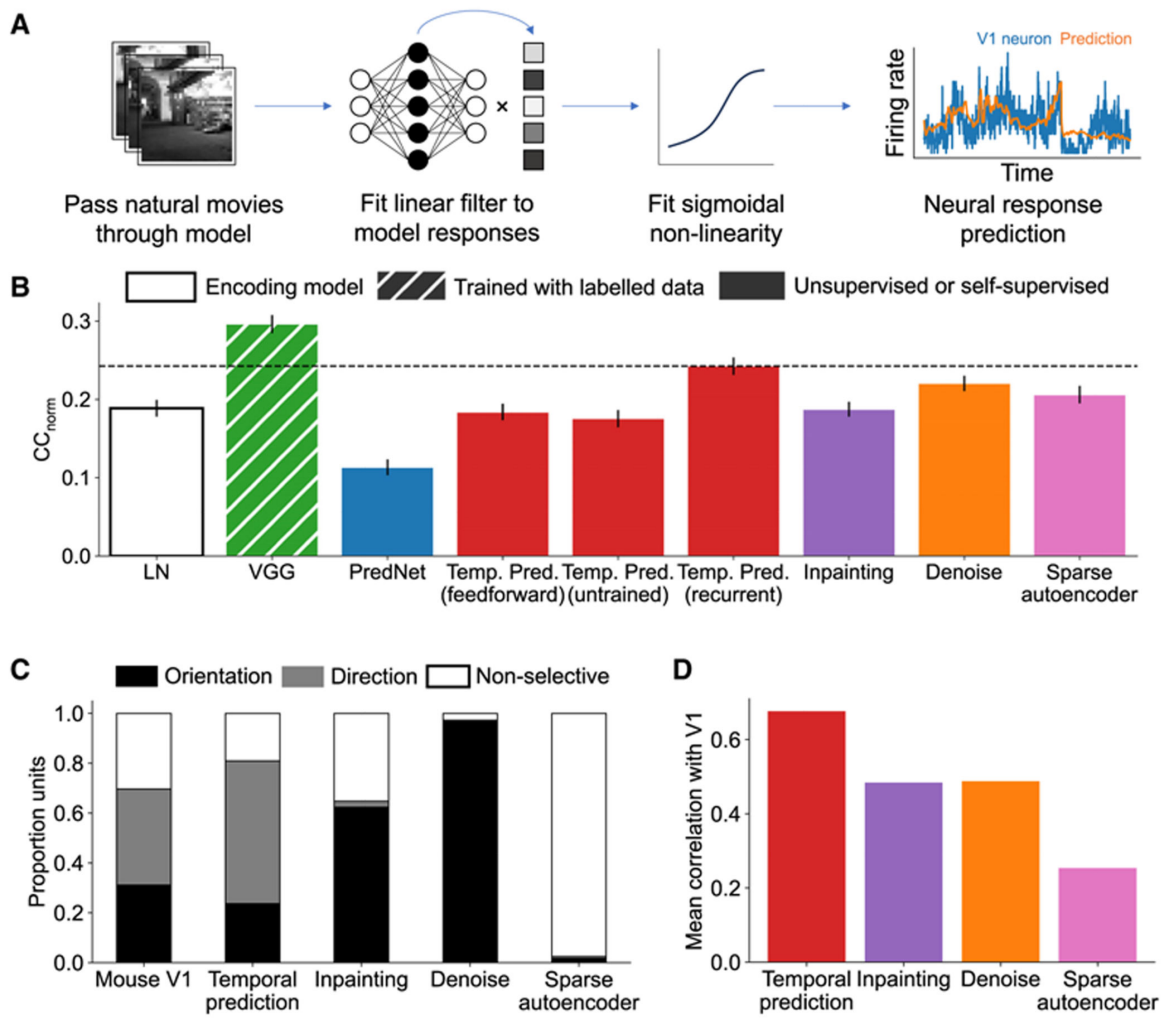
Comparison of neural prediction performance and model connectivity to V1 across alternative models (A) Schematic of the neural response fitting procedure. (B) Performance (average CC_norm_) of the recurrent temporal prediction model (dashed line) relative to other comparison models in predicting neural responses to natural movies. Error bars indicate s.e.m. (C) Distribution of orientation-, direction-, or non-selective units across mouse V1 and each model. (D) Comparison for each model of the average correlation of the functional connectivity profiles (Figures 2B, 2C, 2F, 2G, 2I, and 2J) with those found in mouse V1. A higher correlation indicates an overall better fit with the V1 data. Note that VGG-19 and PredNet could not be included in the functional connectivity analysis. See also Figure S5.

To assess how well temporal prediction performed as a normative model of mouse V1, we examined how well it captured the properties of V1 neurons compared with several other commonly used models (see STAR Methods). First, to directly compare each model’s learned representations with V1, we used regression to predict the responses of awake mouse V1 neurons from the model’s hidden unit activity for two natural movie clips^17^ (Figure 4A). Second, we quantitatively compared model unit properties and connectivity biases to those found in V1. Overall, the recurrent temporal prediction model predicted the recorded neural responses well (mean normalized correlation coefficient [CC_norm_] = 0.242) compared with the other models tested (Figures 4B and 4C) and best accounted for V1 connectivity motifs (Figure 4D).

The linear-nonlinear baseline model consisted of the same fitting procedure as the other models surveyed but was applied directly to the input stimuli and performed significantly worse than the temporal prediction model in predicting V1 responses (CC_norm_ = 0.189, *t*(738) = −6.03, *p* < 0.0001).

VGG-19 and PredNet are both published models that capture elements of V1 responses. VGG-19 is a deep feedforward network trained for object recognition,^34^ while PredNet implements a form of predictive coding.^35^ PredNet was similarly trained for next-frame prediction but, unlike temporal prediction, explicitly uses prediction errors during the inference process.^36^ The temporal prediction model also exceeded the performance of PredNet (mean CC_norm_ = 0.113, *t*(738) = −8.97, *p* < 0.0001) but performed significantly worse than VGG-19 (mean CC_norm_ = 0.296; *t*(738) = 5.40, *p* < 0.0001). However, as a supervised model, VGG-19 is fitted using human-annotated image labels, providing it with constraints not available to other models. Thus, the model is in some senses descriptive rather than normative and not directly comparable to the other models, which were unsupervised.

We also considered two variants of the temporal prediction model, comparing an untrained network with random weights and a feedforward temporal prediction model. In both cases, there was a significant benefit from both training (untrained mean CC_norm_ = 0.175, *t*(738) = −7.03, *p* < 0.0001) and the addition of recurrency (feedforward mean CC_norm_ = 0.184, *t*(738) = −5.74, *p* < 0.0001), implying the importance of these features for modeling V1 neural responses.

Finally, we considered three models based on the same recurrent network architecture but trained using alternative learning objectives. The denoising network aimed to recover the original current frame from noise, the inpainting network aimed to predict the complete current frame given inputs with patches blanked out, and the sparse autoencoder aimed to reproduce the current frame while minimizing hidden unit activity. The recurrent temporal prediction model performed significantly better than the inpainting network (mean CC_norm_ = 0.187, *t*(738) = −4.09, *p* < 0.0001) and the sparse autoencoder network (mean CC_norm_ = 0.206, *t*(738) = −2.31, *p* = 0.021), and non-significantly better than the denoising network (mean CC_norm_ = 0.222, *t*(743) = −1.63, *p* = 0.110), highlighting the importance of the training objective over model architecture alone.

The distribution of orientation- and direction-selective model units varied substantially among these models (Figure 4C). The temporal prediction model most closely reproduced the overall distribution of unit types found in mouse V1,^17^ with comparable proportions of orientation-selective (V1 = 31%, temporal prediction = 24%), direction-selective (V1 = 39%, temporal prediction = 57%), and non-selective units (V1 = 30%, temporal prediction = 19%), albeit with an overrepresentation of direction-selective units. By contrast, the denoising and inpainting networks had a clear overrepresentation of orientation-selective units (62% and 97%, respectively) relative to mouse V1. Similarly, far fewer units met the criteria for direction selectivity across these alternative models, implying that their learned representations had no or only a weak temporal component.

In tandem, we compared how well each model recapitulated the connectivity biases found in mouse V1 (Figure 4D). For each model, we calculated a model connectivity score as the average correlation between the model and V1 connectivity distributions (Figures 2B, 2C, 2F, 2G, 2I, and 2J). Overall, the temporal prediction model (mean = 0.68) had a much closer correspondence to the connectivity profiles found in mouse V1 compared with the other models (in-painting mean = 0.48, denoising mean = 0.49, sparse autoencoder = 0.25). We found no significant correlation between each model’s connectivity score and its neural prediction performance (CC_norm_; *r* = 0.602, *p* = 0.398). Thus, the capacity of a model to predict neural responses in V1 does not imply that it can accurately capture the underlying organization of cortical connectivity.

We additionally compared the learned connectivity of the PredNet model where possible (Figure S5). Although the model’s structure precluded analysis of connectivity motifs dependent on visual space or excitatory and inhibitory subpopulations, we were able to analyze for the motifs described by Ko et al.^3^ and found that these were not well captured by PredNet. However, we were not able to look at lateral connectivity in the VGG model as it lacks recurrency. Hence, due to the limited or lacking lateral connectivity of the VGG and PredNet models, we could not calculate their connectivity scores.

### Variants of the temporal prediction model

We further explored how different modeling parameters impacted the capacity of the temporal prediction model to predict V1 connectivity.

**Figure 5:**
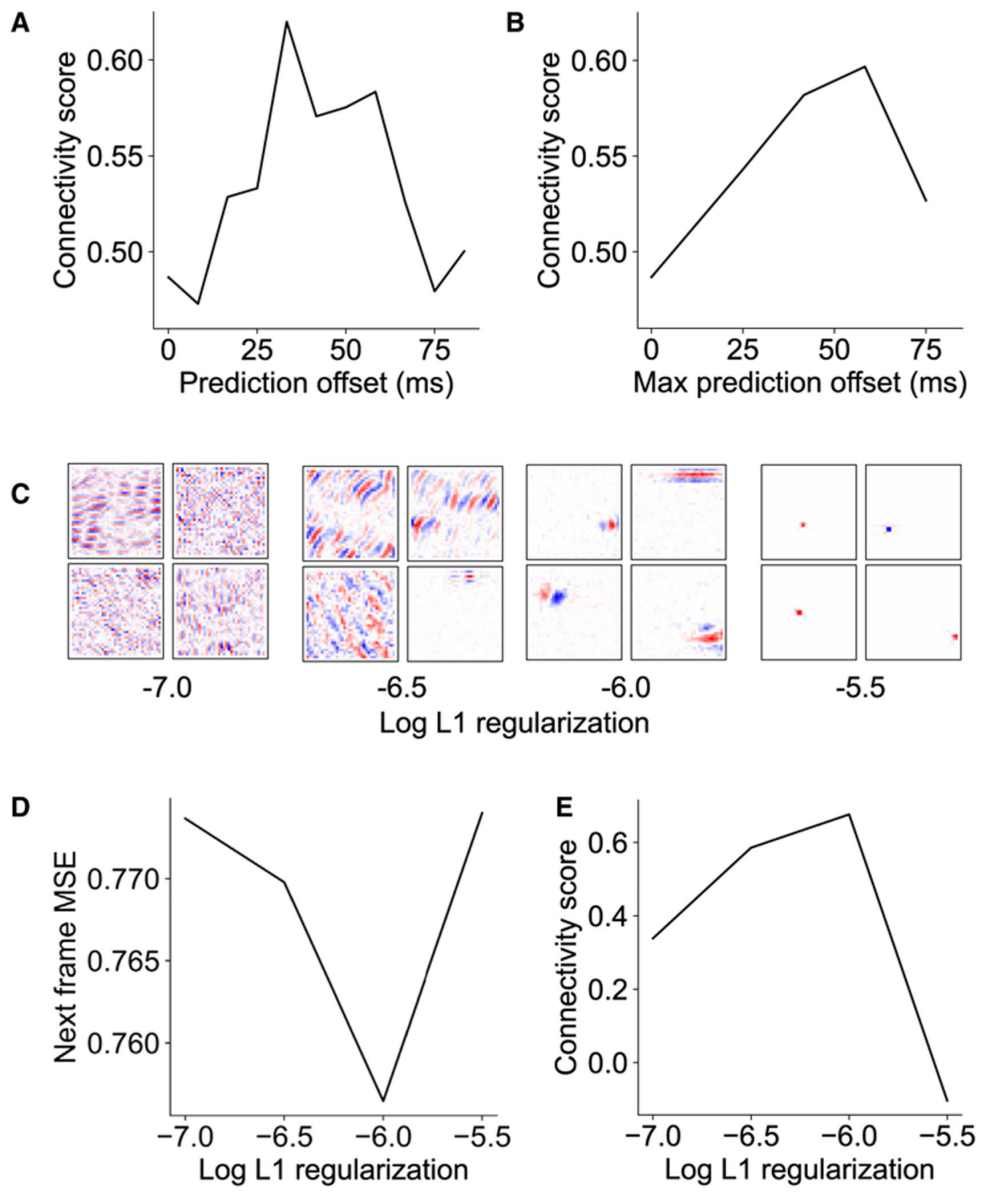
Variants of the temporal prediction model (A) The connectivity score varies as a function of the future prediction offset, with a maximum value reached when predicting the next frame at 33 ms into the future. (B) Connectivity score as a function of increasing future prediction span. (C and D) Example unit receptive fields (C) and next-frame prediction mean squared error (D) as a function of L1 regularization. The optimal V1-like receptive fields coincide with the L1 regularization value that minimizes the mean squared error in the test set. (E) Connectivity score as a function of the L1 regularization value, with the maximum again occurring at the optimal L1 regularization value that minimizes the mean squared error. See also Figure S6.

We first investigated how the future prediction offset affected the model’s connectivity motifs. Using a high frame-rate (120 Hz) dataset,^37^ we produced a continuum of models trained to predict 0–10 frames (0–83 ms) into the future. As expected, we found that the aggregate connectivity score increased smoothly as a function of the future offset, to a maximum of 0.62 at 33 ms (Figure 5A). Similarly, if we trained the model to predict a span of offsets (i.e., the future frames at 0, 0–25, 0–45 ms, etc.), we found that the connectivity score similarly increased with the future offset, with a maximum score of 0.60 when predicting 0–58 ms into the future (Figure 5B). Notably, for the offset-span model, we found that units that integrated information further back into the past tended to project further into the future (*r* = −0.15, *p* < 0.0001; Figure S6). Thus, across these networks, as the prediction target was shifted away from an intermediate temporal offset, the connectivity score declined.

Finally, we investigated the role of the model’s wiring constraint (L1 regularization). Varying L1 regularization had a marked effect on receptive field structure (Figures 5C and 5D). At the highest regularization strength, receptive fields were reminiscent of the center-surround organization of retinal receptive fields, while at weaker regularization strengths, receptive fields were only weakly spatially localized. Only at the optimal regularization strength for next-frame prediction did receptive fields resemble the Gabor-like structure of V1 neurons. A similar dependence on regularization strength was found for the aggregate connectivity score (Figure 5E), where the connectivity score peaked at the optimal L1 regularization strength, declining across neighboring strengths. That the most future-predictive hyperparameter setting is also the most brain-like provides further evidence for temporal prediction as a principle of neural sensory processing.

### Model connectivity supports temporal prediction

**Figure 6:**
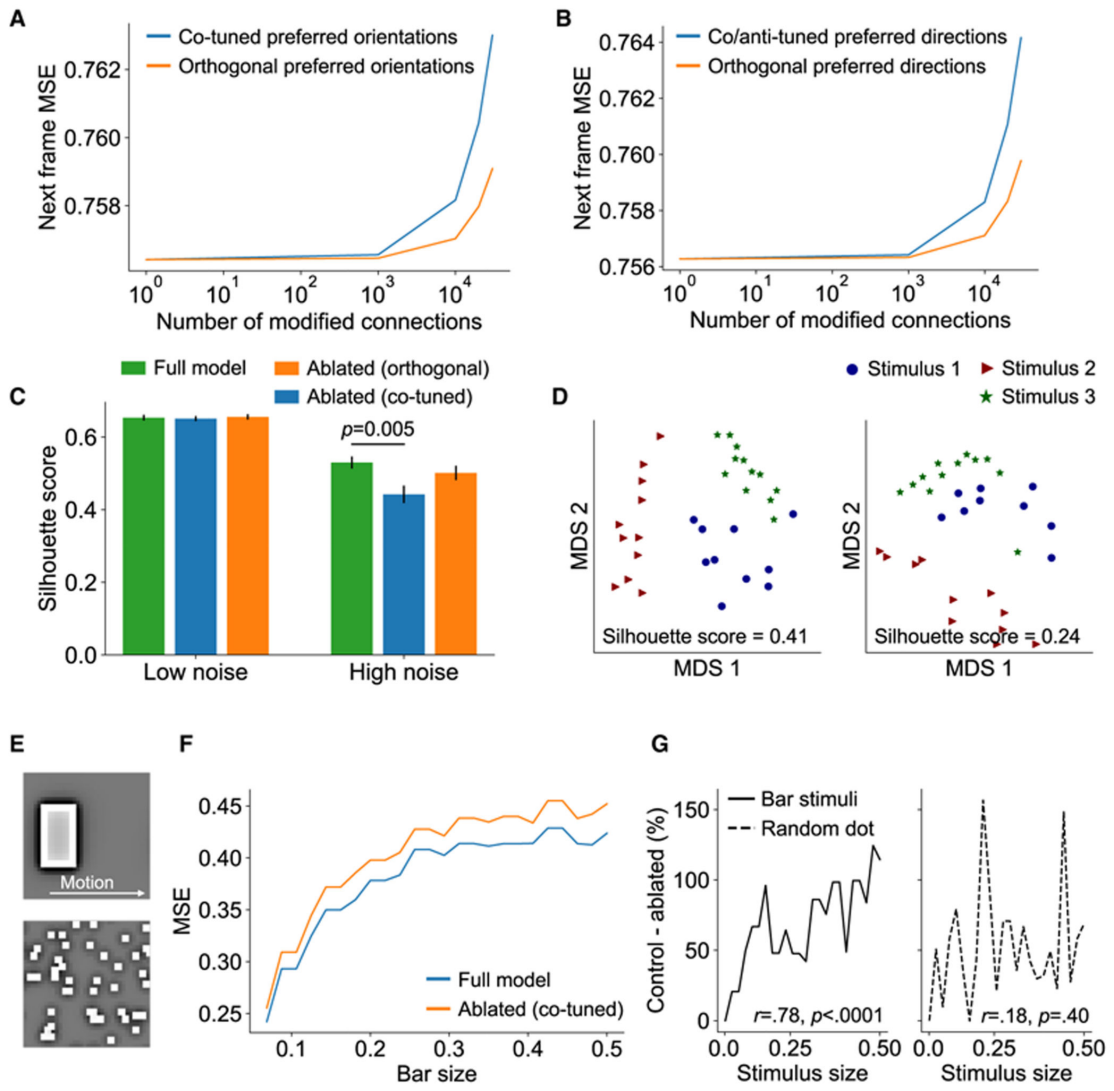
Model connectivity motifs support temporal prediction (A and B) Ablating connections between connected units with similar preferences for stimulus orientation (co-tuned units) or similar and opposite preferences for direction of motion (co/anti-tuned units) impairs next-frame prediction more than for units with orthogonal orientation or direction tuning (orthogonal units). (C) Clustering between network representations (measured via the silhouette score) is significantly lower under high noise when connections between pairs of units that are co-tuned (for orientation) are ablated. This suggests that this connectivity motif may improve the robustness of cortical representations of visual stimuli in noise. Error bars indicate s.e.m. (D) Example plots of unit network activity projected onto two dimensions using multidimensional scaling. The silhouette scores illustrate the clustering across different stimulus types in the default network (left) or the network with ablated connections between co-tuned units (right). (E) Illustration of the moving bar or moving random dot stimuli used to probe the network’s next-frame prediction performance across different stimuli when disrupting specific connectivity motifs. (F) The mean squared error for next-frame prediction in the ablated network increases relative to the control network as a function of bar length. (G) Stimulus size, equated across both stimulus types, predicts the ablation deficit (the percentage increase in mean squared error for the ablated versus control networks) for bar but not random dot stimuli.

To determine how the observed motifs are related to temporal prediction, we perturbed the network’s orientation- and direction-dependent connectivity while measuring next-frame prediction performance (Figures 6A and 6B). To that end, we ablated (set to the median weight) an increasing number of connections in the model across different functional classes of units while measuring next-frame prediction performance. Given the finding in both V1 and the model that similarly tuned units are more likely to be connected, we hypothesized that ablating connections between units with similar orientation or direction tuning (“co-tuned” units for orientation-selective units; “co/anti-tuned” units for direction-selective units) would result in a larger impairment in prediction performance than for units with a large orientation difference or more orthogonal difference in direction preference (“orthogonal” units).

In support of our hypothesis, as the number of ablated connections increased, there was a relatively greater increase in the error when ablating connections between co-tuned and co/anti-tuned versus orthogonally tuned units (orientation: *t*(1,998) = 290, *p* < 0.0001; direction: *t*(1,998) = 382, *p* < 0.0001). Thus, ablating the connectivity motifs in the model described by Ko et al.^3^ specifically impaired temporal prediction.

From a more mechanistic perspective, we investigated how connectivity motifs may impact the network’s internal representations. Considering the finding that units with similar orientation preferences are more likely to be connected, we hypothesized that this connectivity motif may help improve the discriminability of network representations. Within both the visual pathway and deep neural networks, one outcome of processing is to transform representations to facilitate linear readout to downstream regions.^38^ In the case of next-frame prediction, transforming these representations—for example, to increase their separability—could facilitate model performance by helping to decompose visual inputs and thereby better capture their underlying causes.

To test this hypothesis, we presented oriented grating stimuli with different levels of noise while measuring how distinctly the network represented these stimulus classes in low-dimensional space. We used the silhouette score to measure clustering in the model’s hidden activity^39^ and compared these scores between the default and ablated networks (Figures 6C and 6D). Under low-noise conditions (0 dB SNR), we did not find any difference in the silhouette score between the full and ablated networks (cotuned: *t*(46) = 0.240, *p* = 0.814; orthogonal: *t*(46) = −0.186, *p* = 0.853). However, under high-noise conditions (−9.5 dB SNR), a higher silhouette score (indicating improved clustering) was found for the full network compared with the co-tuned but not orthogonal ablated networks (co-tuned: *t*(46) = 2.94, *p* = 0.005; orthogonal: *t*(46) = 1.09, *p* = 0.282). Thus, under conditions of high noise, connections between similarly orientation-tuned units may help disentangle the internal representations of different stimuli.

Finally, we examined the potential roles of the connectivity motifs described by Iacaruso et al.^1^ in temporal prediction. We hypothesized that the tendency of V1 neurons to synapse with neurons of similar orientation tuning in co-axial space—that is, when their receptive fields are aligned in visual space—might facilitate the detection of contiguous moving edges. This should aid the network in next-frame prediction.

Under this hypothesis, we predicted that ablating connections between these model units would impair the next-frame prediction of contiguous-edge-like features (i.e., a moving bar). Furthermore, this impairment should increase with the bar length, under the assumption that this motif detects elongated features. As a control, we compared the bar stimulus with moving random dot stimuli, equating the total area of both (Figure 6E). Since random dot stimuli are non-contiguous, increasing the stimulus area (unlike bar length) should not affect the degree of impairment produced by ablating model connectivity. In line with this hypothesis, we found that stimulus area was correlated with the impact of ablations on mean squared error for the moving bar stimuli (Figure 6F; *r* = 0.778, *p* < 0.0001) but not random dot stimuli (Figure 6G; *r* = 0.180, *p* = 0.400).

Together, these results suggest that the identified connectivity motifs have specific functions in supporting temporal prediction.

## Discussion

The recurrent temporal prediction model exhibits response properties and functional connectivity patterns remarkably akin to those found in mouse V1, providing a unifying normative explanation for these wiring biases. In particular, the model captured the relationship between both short- and long-range connectivity patterns and neuronal preferences for stimulus orientation and direction of motion, as well as spatial differences in the inputs from excitatory and inhibitory cells to direction-selective cortical neurons.

The extent to which cortical circuits are fundamentally stereo-typed remains an enduring question in systems neuroscience. The concept of a canonical microcircuit proposes that cortical networks follow the same basic organization in which functional differences are defined primarily by their inputs and outputs, rather than by idiosyncratic, local circuits.^40,41^ In support of this hypothesis, recurring cortico-thalamic and cortico-cortical loop motifs, as well as cell-type- and layer-specific patterns of connectivity, have been found to be consistent across many cortical areas.^42,43^ However, the extent to which cortex-wide connectivity motifs might relate to the functional properties of cortical neurons is still emerging.^44^

Within mouse V1, our study demonstrates that a plausible computational principle—temporal prediction—can account for these functional connectivity patterns. Crucially, the network model was not optimized for specific response properties of visual neurons (e.g., particular receptive field characteristics). Instead, the resulting patterns of connectivity arose naturally as an emergent function of optimizing for the more general objective of predicting the neurons’ future inputs. These results suggest that wiring biases found in mouse V1 are not arbitrary but rather that they underpin an important cortical function.

### Comparison to other normative models

Despite the clear functional importance of recurrent connectivity in V1, there are comparatively few normative modeling studies addressing this topic. The key contribution of the present work is in uniting different aspects of both short-range and long-range V1 connectivity with neuronal feature preferences under a single unsupervised learning objective.

Sparse coding networks have been widely employed in modeling receptive fields and response properties^16,45,46^ and more recently have been applied to local connectivity in the visual cortex. Sparse coding argues that the brain is optimized to represent stimuli efficiently such that only a small number of neurons are strongly activated at a given time. When trained on static images, sparse coding models have been found to replicate the like-for-like connectivity pattern among units with similar orientation tuning.^47,48^ Where motion has been included in these models, they can capture the asymmetry in excitatory and inhibitory inputs for direction tuning.^49^ However, these sparse coding models have been shown to replicate simple-cell responses only, but not complex-cell responses. Similarly, such models have not been shown to reproduce other distinct connectivity profiles across separate excitatory and inhibitory cell populations or the long-range tuning biases reported in this study.

Finally, local recurrent connectivity in V1 has also been approached from a Bayesian perspective, where the dependence of cortical connectivity on the similarity in orientation tuning is argued to represent an optimal means of integrating contextual information.^50^ However, this Bayesian model depends on hard-coded basis functions derived from V1 simple cells, and unlike our approach, it can neither be said to be truly unsupervised nor learned exclusively from natural stimulus statistics.

In the context of this study, we found that only the temporal prediction model could closely reproduce the observed relationships between V1 neurons and their functional connectivity. Thus, the results cannot be accounted for by the choice of dataset or model architecture but are specific to the temporal prediction model’s training objective. The temporal prediction model therefore provides a more complete explanation than the other models for the relationship between the connectivity of visual cortical neurons and the stimulus features to which they are tuned. In turn, these results suggest thatthe functional specificity of connections in V1 enables the brain to process dynamic stimuli by facilitating the prediction of upcoming sensory information. These predictions are critical for guiding complex actions,^9,10^ such as those required to catch a moving prey or, in the case of a tennis player, to return the ball, which depend on estimating the future state of the world.

### Comparison to the biology

While the current temporal prediction model is trained using backpropagation through time, the principle of temporal prediction itself is largely agnostic to the underlying learning mechanisms. Indeed, novel and more biologically plausible learning algorithms are being developed that could, in principle, be applied to learn temporal prediction for the current network.^51,52^ In this sense, the present work does not preclude either a hard-wired or learned origin for the connectivity patterns found in visual cortex.^6,7^

From an evolutionary perspective, temporal prediction is likely to confer several advantages. By encoding only those features that are efficiently predictive of future sensory inputs, temporal prediction provides a principled way of extracting underlying variables and discarding non-predictive, and therefore less behaviorally relevant, information.^9^ Furthermore, given the inherent delays due to neural conduction and processing in sensory pathways and in preparing motor outputs, some form of predictive processing may be essential to accurately guide an animal’s actions.^10^

Given that the model’s structure is learned from an initial random state, such a configuration can, at least in theory, emerge from the interplay of some optimization principle and the natural statistics of visual inputs. Following the onset of vision, the connectivity of mouse V1 neurons that respond to similar visual features progressively increases.^53^ These response-specific connectivity patterns still develop in dark-reared mice, indicating that the emergence of like-for-like wiring biases is not dependent on visual experience.^54^ Nevertheless, the relationship between connection probability and the similarity of V1 responses to natural movies (but not the similarity of their orientation preferences) was found to be weaker in dark-reared mice than in animals reared with normal visual inputs. Thus, it is likely that these biases in functional connectivity result from an interplay of innate developmental programs that, at least to some extent, are later fine-tuned by sensory experience.^55^ Such learning might then require some neurons or neuronal compartments to represent prediction or prediction errors congruent with the temporal prediction model output units and loss function. Notably, the hidden units in the temporal prediction model sometimes developed mixed sensitivity to both visual stimuli and prediction errors, which is consistent with cortical circuits in the brain where neurons rarely exist in a pure error-coding capacity.^25,31^

In conclusion, we show that many aspects of functional connectivity in mouse V1 can be parsimoniously described by a single framework—temporal prediction. By optimizing a recurrent network for temporal prediction, model units naturally recapitulate both structural and functional properties of mouse visual cortex. Consequently, even seemingly disparate examples of connectivity rules may be united by a simple underlying principle of cortical organization.

## Resource Availability

### Lead contact

Further information and requests for code or data should be directed to Andrew J. King (andrew.king@dpag.ox.ac.uk).

### Materials availability

This study did not generate new unique reagents.

## STAR⋆METHODS

Detailed methods are provided in the online version of this paper and include the following:

- KEY RESOURCES TABLE
- EXPERIMENTAL MODEL AND SUBJECT DETAILS
- METHOD DETAILS
  ○ Dataset
  ○ Network model
  ○ Implementation
  ○ Comparison models
  ○ Neural response predictions
- QUANTIFICATION AND STATISTICAL ANALYSES
  ○ Receptive field mapping
  ○ Unit inclusion criteria
  ○ Unit tuning characteristics
  ○ Natural movie response correlations
  ○ Prediction errors analyses
  ○ Unit connectivity
  ○ Visual space-dependent connectivity
  ○ Ablation experiments
  ○ Control analyses
  ○ Mouse V1 comparisons data

## Star⋆Methods

### Key Resources Table

**Table:** 

REAGENT or RESOURCE	SOURCE	IDENTIFIER
Biological samples		
Allen Institute Visual Coding database	The Allen Institute	https://portal.brain-map.org/circuits-behavior/visual-coding-neuropixels
Software and algorithms		
Analysis scripts	This paper	https://github.com/sebbkw/temporal_prediction_connectivity
Python	https://www.python.org/	N/A
PyTorch	https://pytorch.org/	N/A

## Experimental Model and Subject Details

Experimental data used for neural response prediction was taken from the Allen Institute for Brain Science Neuropixels Visual Coding dataset, whose detailed experimental procedures have been published elsewhere.^17^ The dataset used for neural fitting was comprised of recordings from 15 male wildtype C57BL6/J mice (*n* = 739 neurons).

## Method Details

### Dataset

Training data consisted of natural wildlife videos using the same dataset as described previously.^6^ Videos were taken from the repository http://www.arkive.org/species and contributed by: BBC Natural History Unit, http://www.gettyimages.co.uk/footage/bbcmotiongallery; BBC Natural History Unit & Discovery Communications Inc, http://www.bbcmotiongallery.com; Granada Wild, http://www.itnsource.com; Mark Deeble & Victoria Stone Flat Dog Productions Ltd., http://www.deeblestone.com; Getty Images, http://www.gettyimages.com; National Geographic Digital Motion, http://www.ngdigitalmotion.com. In brief, videos were converted to grayscale, bandpass filtered then downsampled to 180x180 pixels. Finally, each video was cropped into non-overlapping 36x36 pixel patches of 50 frames each, leading to a total of 40,000 clips for the training dataset and 4,000 clips for the validation dataset used for hyperparameter selection. In addition, to mimic the effects of noise present in the nervous system, Gaussian noise was added to each video clip during training with a signal-to-noise ratio of 6 dB.^6^

For the models in which the temporal offset was varied, training data consisted of a set of 120 Hz naturalistic videos^37^ (available at https://figshare.com/articles/dataset/Natural_movies/24265498). For each temporal offset, for the target to be predicted, but not for the input, we shifted the 120 Hz videos 0-10 frames (0-83 ms) into the future. Both the input and target datasets were then down-sampled to 24 Hz to approximately match the 25 Hz frame rate of the original temporal prediction model, and then cut into 50-frame long clips. This setup ensured that the input dataset was the same across temporal offsets, while only the frame targets varied as a function of temporal offset.

### Network model

The model was implemented as a single-layer recurrent network with a linear readout layer to project the hidden activity to the network’s output predictions. The network’s input consisted of a 50-frame video clip, where the model was trained to predict each subsequent frame given the preceding video frames in the clip. More formally, the network receives a 1,296 length vector **u**[*t*] at each time step *t*, consisting of the flattened 36x36 pixel video frame. The 2,592 length hidden state vector **s**[*t*] at each time step *t* is then given by:

$$s[t]=\text{f}\left(W_{\text{in}}u[t]+W_{\text{rec}}s[t-1]+b_{\text{rec}}\right)$$

where f is the ReLU function, **W**_in_ is the weight matrix which describes the input weights to the network, **W**_rec_ is the weight matrix which describes the hidden, recurrent weights mapping the previous state s[*t* − 1] to the new hidden state **s**[*t*], and **b**_rec_ is the bias term.

The hidden activity vector s[*t*] at each time step *t* is then mapped to the output predictions $\hat{v}[t]$ by:

$$\hat{v}[t]=W_{\text{out}\;}s[t]+b_{\text{out}\;}$$

where **W**_out_ is the weight matrix describing the linear mapping from the hidden state to the output prediction and **b**_out_ is the bias term for the output weights.

In addition, to enforce Dale’s Law whereby hidden units make exclusively excitatory or inhibitory connections, each recurrent weight *w* was constrained during the forward pass as:

$$w\leftarrow \{\begin{array}{l} -|w|\;\text{if}\;\text{inhibitory}\;\\ +|w|\;\text{if}\;\text{excitatory}\; \end{array}$$

with a total of 2,332 (90%) units set as excitatory and the remaining 260 (10%) as inhibitory units.^56,57^

The network was then optimized using backpropagation to minimize the loss function *E*:

$$E=\sum_{n=1}^{N}\sum_{t=1}^{T}∥{\hat{v}}_{n}[t]-v_{n}[\mathrm{t+1}]{∥}_{2}^{2}+\lambda \left(∥W_{\text{in}}{∥}_{1}+∥W_{\text{rec}}{∥}_{1}+∥W_{\text{out}}{∥}_{1}\right)$$

where *n* is the clip number, *N* is the total number of clips in a minibatch, *T* is the total number of time steps, and ${\hat{v}}_{n}[t]-v_{n}[t+1]$ is the difference between the predicted pixel values ${\hat{v}}_{n}[t]$ and true future pixel values **v**_*n*_[*t* + 1]. Finally, L1 regularization is included as the sum of absolute values of all weights in the network, weighted by the λ hyperparameter.

### Implementation

The temporal prediction model was implemented in PyTorch, with gradient descent performed using the ADAM optimizer set at a learning rate of 10^−4^. Unless otherwise noted, the regularization strength hyperparameter λ was set at 10^-6^ after a hyperparameter search across lambda values (λ range = 10^-5.5^ - 10^-7^) to minimize the mean squared error on the held-out validation set.

### Comparison models

The inpainting, denoising and sparse autoencoder networks consisted of the same network architecture as the recurrent temporal prediction model but with modified datasets and training objectives. For the inpainting network, the input dataset was masked with 8 randomly placed 8x8 pixel patches on each frame. For the denoising network, the input was combined with Gaussian noise with a signal-to-noise ratio of 3 dB. Finally, the sparse autoencoder was trained on the same dataset as the temporal prediction model but was trained to recover the current frame under a sparsity constraint. In all these networks, the models were optimized to produce the unmodified current frame (rather than subsequent frame, as for the temporal prediction model) by minimizing the mean squared error between the predicted and actual current frame:

$$E=\sum_{n=1}^{N}\sum_{t=1}^{T}∥{\hat{v}}_{n}[t]-v_{n}[t]{∥}_{2}^{2}+\lambda \left(∥W_{\text{in}}{∥}_{1}+∥W_{\text{rec}}{∥}_{1}+∥W_{\text{out}}{∥}_{1}\right)$$

For the sparse autoencoder, an additional regularization term λ_act_ was included as the absolute sum of activity across all units, to encourage sparsity in the network’s representations:

$$E=\sum_{n=1}^{N}\sum_{t=1}^{T}∥{\hat{v}}_{n}[t]-v_{n}[t]{∥}_{2}^{2}+\lambda \left(∥W_{\text{in}}{∥}_{1}+∥W_{\text{rec}}{∥}_{1}+∥W_{\text{out}\;}{∥}_{1}\right)+\lambda_{\text{act}\;}\sum_{n=1}^{N}\sum_{t=1}^{T}{∥s}_{n}{[t]∥}_{1}$$

For the inpainting and denoising networks, the L1 weight regularization hyperparameter was chosen as for the temporal prediction network to minimize the mean squared error on the validation set across a range of values. For the sparse autoencoder, where no such comparable selection criterion exists, the hyperparameter set was qualitatively optimized to produce the most biologically realistic receptive fields.^7^

For VGG-19, we used a publicly available model from the PyTorch ‘torchvision.models’ package, pre-trained on the ImageNet dataset for object recognition.^34^ For each layer of the network, we took the hidden activity as the concatenated, flattened feature maps of each filter in the layer.

For PredNet, we reimplemented the model described by Lotter et al.,^35^ trained for next-frame prediction on the same dataset as the main temporal prediction model. However, because the resolution halves at each layer, we used a slightly larger input size of 40x40 pixels. Again, as for VGG-19, we took the hidden activity across layers as the concatenated, flattened feature maps of each filter in that layer.

The LN model used the same basic fitting procedure as the other models. However, whereas in the other models used to predict neural responses we regressed the neural responses on each pre-trained network’s hidden activity, the output of the LN model was directly fitted to the neural responses.

### Neural response predictions

Neural data were taken from the Allen Institute’s Neuropixels Visual Coding dataset.^17^ For each model, a linear-nonlinear mapping was fitted to predict the response of V1 units to natural movie stimuli (“Natural Movie One” and “Natural Movies Three”, 150 seconds total) from the pre-trained model’s hidden unit activity. We divided the dataset into 15x10 second clips, taking 3 representative clips (30s total) as the final held-out test set, with the remaining 120 seconds taken as the training and validation sets with hyperparameter selection by k-fold cross-validation. We included all recorded V1 units from wildtype mice whose noise-to-signal power ratio^58^ in response to the natural movies was below 60.

For each model, the neural fitting process consisted of first fitting a linear mapping using Lasso regression before fitting a rectified sigmoidal non-linearity.^59^ Prior to fitting, the dimensionality of the model unit activity that was regressed on was reduced to the first 200 components of a principal component analysis (PCA) fitted on the training-set model responses. PCA was used to equate the number of parameters across models and increase the efficiency of model fitting.^7^ The non-linearity $\hat{r}(x)$ was defined as:

$$\hat{r}(x)=\text{ReLU}\left(\frac{a}{1+e^{\frac{c-x}{b}}}+d\right)$$

where the parameters *a, b, c* and *d* were optimized to minimize the mean squared error between the true and predicted neural firing rate using the SciPy “curve_fit” function.^60^ The L1-regularization strength (α) of the Lasso was chosen via cross-validation from 40 values log-spaced between 10^1^ and 10^-5^ to maximize the average normalized correlation coefficient (CC_norm_)^61,62^ across each fold’s validation set for the combined linear-nonlinear mapping. The reported values are finally taken as the performance on the held-out test set.

## Quantification and Statistical Analyses

### Receptive field mapping

Model unit receptive fields were estimated using their response-weighted average. In brief, the responses of model units to 25,000 frames of random Gaussian noise (μ=0, σ=1) were produced. Each noise frame was then weighted by the unit’s response to give the receptive field estimate. Model receptive fields were subsequently parameterized by a Gabor function to extract the receptive field centers and 2D extent.

We calculated the unit’s spatiotemporal response-weighted average similarly to the standard response-weighted average, but included the past 7 frames. The temporal power was then taken as the mean squared value over space for each time step, normalized by the total power. Similarly, to calculate the projective center of mass, we took the mean squared weight for each unit for each predicted future-frame time step. For each of these, we then determined the center of mass as the average of the time values weighted by that time step’s corresponding normalized power.

### Unit inclusion criteria

To maintain consistency across analyses, only those units whose receptive fields that were spatially well defined and which could be well modeled as Gabors were included for analysis. To that end, units whose receptive fields were less than 0.5 pixels in size and therefore had little spatial extent (19% of total units) or which were poorly fitted by the Gabor function were excluded (r<0.7, 12% of total units; 30% including both criteria). Short-range connections were defined as those less than 15° (2.5 pixels) and long-range as greater than 30° (5 pixels).^1^ Connections greater than 9.17 pixels were excluded because of the experimental constraints imposed by screen size. In the case of Ko et al.,^3^ connections were not explicitly defined according to the distance between receptive fields, but we use the same short-range convention as in Iacaruso et al.^1^ that, under the assumption of retinotopy, physically short-range connections (<50 μm) are likely to be close in visual space.

### Unit tuning characteristics

To measure the model units’ tuning properties, each unit’s response to sinusoidal gratings was recorded. Gratings varied in temporal frequency (0.02-0.25 cycles/frame), spatial frequency (0.03-0.5 cycles/pixel), and orientation (0-360 degrees) with an amplitude of ±1. Each unit’s preferred temporal frequency, spatial frequency and orientation were taken as the parameter or parameter combination that maximized the unit’s mean response across 50 frames. For those analyses dependent on the unit’s spatial location, only units within the central 16x16 pixel bounds of the visual fields were included to avoid edge effects.

Orientation and direction selectivity were quantified as OSI and DSI, respectively:

$$\begin{array}{c} OSI=\frac{R_{\text{pref}}^{\text{Or}}-R_{\text{orth}}^{\text{Or}}}{R_{\text{pref}}^{\text{Or}}+R_{\text{orth}}^{\text{Or}}}\\ DSI=\frac{R_{\text{pref}}^{\text{Dir}}-R_{\text{opp}}^{\text{Dir}}}{R_{\text{pref}}^{\text{Dir}}+R_{\text{opp}}^{\text{Dir}}} \end{array}$$

where $R_{\text{pref}\;}^{\text{Or}}$ and $R_{\text{orth}}^{\text{Or}}$ are the unit responses at the preferred and orthogonal orientations, and $R_{\text{pref}\;}^{\text{Dir}\;}$ and $R_{\text{opp}}^{\text{Dir}}$ are the unit responses at the preferred and opposite (+180 degrees) directions. For Figure 2, we take the same thresholds as Ko et al.,^3^ where direction selective units were defined as those with OSI values exceeding 0.4 and DSI values exceeding 0.3. For Figure 3, where no threshold is given in Rossi et al.,^5^ we take the more stringent threshold of 0.8 for direction-selective units.

Model units were classified as simple- or complex-like based on their phase-responsiveness to drifting grating stimuli. Quantitatively, units with a modulation ratio *F >* 1 were classed as simple-cell-like or as complex-cell-like for a modulation ratio < 1. The modulation ratio was defined as $F=\frac{F_{1}}{F_{0}}$ where *F*_0_ is the mean response of the neuron to its preferred stimulus and *F*_1_ is the amplitude of the fitted sinusoid to the neuron’s response to its preferred stimulus. Where the correlation between the fitted sinusoid and the true response was < 0.9, we did not calculate the modulation ratio for that unit.

### Natural movie response correlations

One hundred 50-frame clips were randomly selected from the validation set and the response recorded for each model unit. The response correlation was then taken as the average correlation across the set of clips for each pair of units in the network.

### Prediction errors analyses

We used two paradigms to assess the presence of sensitivity to prediction error in the network hidden units: oddball stimuli and omission stimuli. In the oddball paradigm, a deviant stimulus interrupts the pattern generated by a preceding set of standard stimuli. In the omission paradigm, the violating stimulus consists of an omission – i.e. the absence of an expected stimulus presentation.

In the odd-ball paradigm, we presented the model with stimuli consisting of two full-field gratings of orientations A and B that each spanned 0°, 45°, 90° or 135° (Figure S2A). The omission paradigm was similar but consisted of a single full-field grating A and a blank stimulus B. A and B stimuli alternated for 25 frames, while the omission or deviant position was varied to occur after 5-25 standard frames. We constructed two sets of control stimuli to ensure that prediction-error responses could not be explained by differences in stimulus tuning or unrelated network dynamics. First, we compared the response to the violating stimulus with that generated by the same set of stimuli using the standard stimulus (i.e. ABABB vs. ABABA). Second, we constructed a ‘shifted’ set of stimuli, where the deviant position was matched but without violating the pattern (i.e. ABABB vs. BABAB).

We chose a relatively conservative set of criteria to avoid miscategorizing non-prediction-error responses. Specifically, a unit was defined as prediction-error-like only if for a particular orientation or orientation pair it had no response in either control condition and responded to the violating stimulus across at least 5 different deviant positions. We also adopted slightly looser criteria (Figure S2C), whereby prediction-error-like responses were defined when responses to deviant or omission stimuli exceeded three times the response to control stimuli.

### Unit connectivity

Units were defined as connected if their connection strength exceeded the 95^th^ percentile of connection weights (W_rec_) across all pairs of units. Due to the sparse nature of the recurrent weight connectivity matrix, this threshold equated to rejecting the very low or zero weight connections, while retaining the smaller subset of highly connected units. Thus, varying this threshold across a range of values (92.5-99 percentile) did not qualitatively change the results.

### Visual space-dependent connectivity

For each presynaptic ensemble, visual space was normalized according to the receptive field center and preferred orientation of the postsynaptic unit. Receptive field centers were first translated such that the postsynaptic unit receptive field was centered at the origin:

$$\left[\begin{array}{c} x_{\text{pre}\;}^{\prime }\\ y_{\text{pre}\;}^{\prime }\\ 1 \end{array}\right]=\left[\begin{array}{ccc} 1 & 0 & -x_{\text{post}\;}\\ 0 & 1 & -y_{\text{post}\;}\\ 0 & 0 & 1 \end{array}\right]\left[\begin{array}{c} x_{\text{pre}\;}\\ y_{\text{pre}\;}\\ 1 \end{array}\right]$$

Next, receptive field centers were rotated according to the postsynaptic unit’s preferred orientation *θ*:

$$\left[\begin{array}{c} x_{\text{pre}\;}^{\prime }\\ y_{\text{pre}\;}^{\prime }\\ 1 \end{array}\right]=\left[\begin{array}{ccc} \text{cos}\left(\theta -\frac{\pi }{2}\right) & -\text{sin}\left(\theta -\frac{\pi }{2}\right) & 0\\ \text{sin}\left(\theta -\frac{\pi }{2}\right) & \text{cos}\left(\theta -\frac{\pi }{2}\right) & 0\\ 0 & 0 & 1 \end{array}\right]\left[\begin{array}{c} x_{\text{pre}\;}\\ y_{\text{pre}\;}\\ 1 \end{array}\right]$$

For the comparison with Iacaruso et al.,^1^ presynaptic units were binned into co-orthogonal and co-axial receptive field centers according to whether they fell in one of the four quadrants orthogonal to or parallel with the postsynaptic unit’s preferred orientation (i.e., defined by *y* = –*x* and *y* = *x*). For the comparison with Rossi et al.,^5^ presynaptic units were binned according to whether they fell opposite to or ahead of the postsynaptic unit’s preferred direction of motion (i.e., defined by *x* = 0). Presynaptic densities were computed by binning the presynaptic receptive field centers (taken as the center of the Gabor fit to the unit’s receptive field, as described above) across visual space.

### Ablation experiments

For the ablation experiments, we randomly selected *n* connections belonging to the relevant class of units. For each of these units, the connection was ‘ablated’ by setting its value to the median connection weight for recurrent weights between the excitatory subpopulation of the network. This was repeated 1,000 times and the average taken to obtain the MSE values in Figure 6.

For the silhouette score analyses, we presented three classes of grating stimuli whose orientations were offset by 0 °, 11.25° and 22.5°, repeating the analysis across the complete span of orientations (0-360°). Over these classes of stimuli, we then calculated the silhouette score to measure the level of distinctness of the three classes in the hidden representations. The silhouette score is bounded between -1 and 1, with a high score indicating that the clusters are well separated according to the stimulus (orientation) class.^39^

### Control analyses

The distribution of preferred orientations and directions among model units was not uniform (Figure S1). To control for the possibility that the observed model connectivity distributions resulted from this overrepresentation of particular orientation and direction tuning preferences, we compared the true model results to those after randomly shuffling across model weights. Specifically, we took the total set of model units fulfilling the relevant criteria for each analysis (e.g. orientation selectivity, receptive field distance, etc.) and randomly shuffled the recurrent weights connecting these units. It is important to note that we used these shuffled weights only for the connectivity analyses, not for any other part of any analysis, such as getting unit responses for response weighted averages. We repeated this process 1,000 times, taking the mean value of the resulting distribution to compare to the true unshuffled model results.

### Mouse V1 comparisons data

Mouse data for comparison were either extracted from published figures using WebPlotDigitizer (Figures 1F and 2J), taken as the exact statistics from the published paper (Figures 2B, 2C, 2F, 2G, and 2I) or computed directly from the Neuropixels Visual Coding dataset from the Allen Institute^17^ (Figures 1C, 1E, 4C, and S1). For the V1 receptive fields (Figure 1C), these were estimated by fitting a linear filter to predict the responses of V1 single units to natural movies as described above.

To compare how well each model captured the connectivity patterns described for mouse V1, we calculated a model connectivity score as the average of the Pearson correlation coefficients between the described neural connectivity profiles (data in Figures 2B, 2C, 2F, 2G, 2I, and 2J) and the corresponding connectivity profiles of the given model.

## Supplementary Material

Supplementary Material

## Data Availability

This paper analyzes existing, publicly available data, accessible at https://portal.brain-map.org/circuits-behavior/visual-coding-neuropixels. Original code used to generate analyses and figures presented in this manuscript has been deposited on GitHub and is publicly available as of the date of publication at https://github.com/sebbkw/temporal_prediction_connectivity. Any additional information required to reanalyze the data reported in this paper is available from the lead contact upon request.

## In brief

Klavinskis-Whiting et al. show that feature-specific connectivity patterns in mouse primary visual cortex are captured by simple recurrent neural networks trained to predict their future inputs. This temporal prediction model accounts for a range of observed connectivity motifs, providing a unifying explanation for cortical functional connectivity.

## Highlights

- Neurons in primary visual cortex show feature-specific connectivity patterns
- Recurrent networks trained for temporal prediction recapitulate this connectivity
- Temporal prediction outperforms models based on alternative training objectives
- The connectivity of early visual cortex may be optimized for temporal prediction
