# Supplementary material for: Prediction of future input explains lateral connectivity in primary visual cortex
Source: Curr Biol. Author manuscript; Available in PMC 2025 Mar 13. (PMC7617481; doi:10.1016/j.cub.2024.11.073)
Supplement: Supplementary Material [file EMS203656-supplement-Supplementary_Material.zip › 1-s2.0-S0960982224016403-mmc1.pdf]

Current Biology, Volume *35*

## Supplemental Information

# Prediction of future input explains lateral connectivity in primary visual cortex

**Sebastian Klavinskis-Whiting, Emil Fristed, Yosef Singer, M. Florencia Iacaruso, Andrew J. King, and Nicol S. Harper**

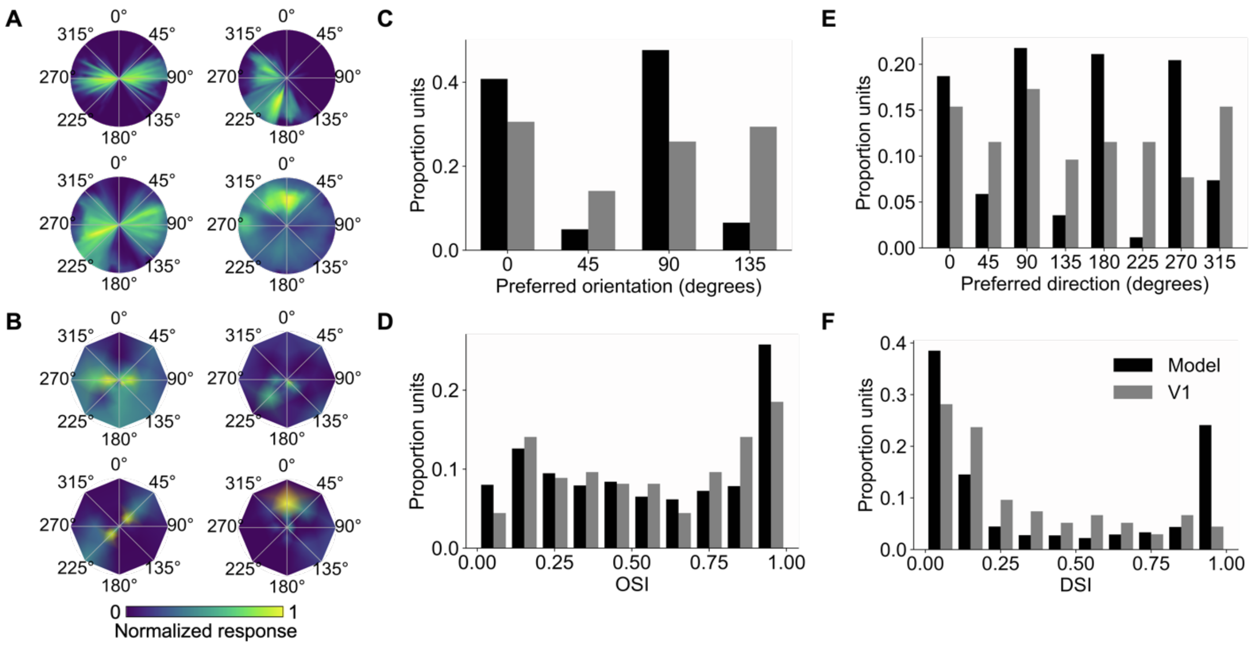


**Figure S1: Model and mouse V1 tuning properties, Related to Figure 1.**
All mouse data from the Allen Brain Institute's visual coding dataset.
**(A)** Example model unit responses to drifting gratings as a function of temporal frequency (1-8 Hz, radial axis) and direction (polar angle).
**(B)** Example mouse V1 responses to drifting gratings as a function of temporal frequency (2-15 Hz, radial axis) and direction (polar angle).
**(C)** Distribution of preferred drifting grating orientations for model units and mouse V1.
**(D)** Distribution of orientation selectivity indices (OSI) for model units and mouse V1.
**(E)** Distribution of preferred drifting grating directions for model units and mouse V1.
**(F)** Distribution of direction selectivity indices (DSI) for model units and mouse V1.

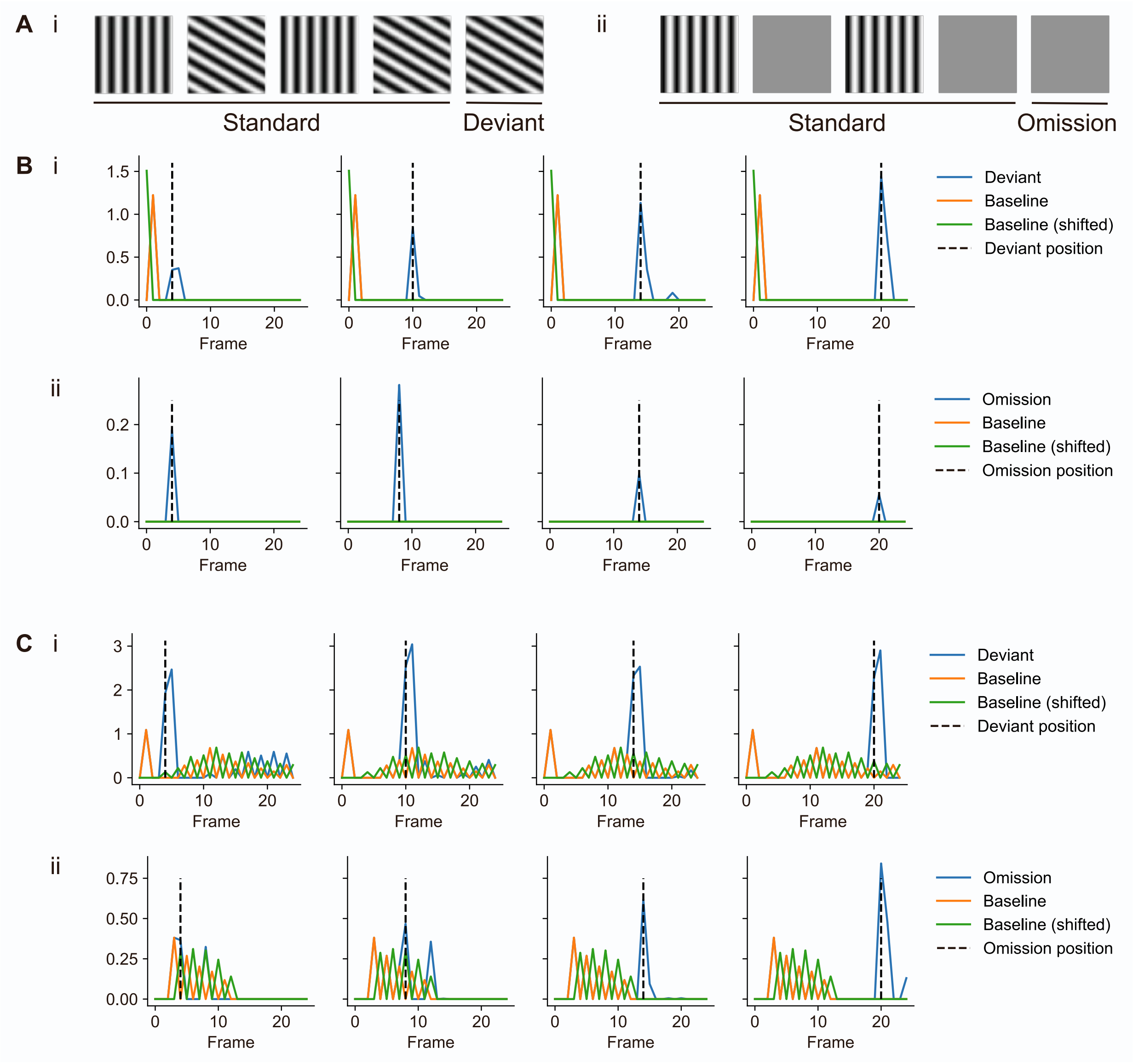


**Figure S2: The recurrent temporal prediction model generates prediction error responses, Related to Figure 1.**
**(A)** Example deviant (i) and omission (ii) stimuli used to probe the network.
**(B)** Example unit activations for deviant (i) and omission (ii) stimuli. Each subplot reflects a different deviant position in the sequence, with the prediction error response tracking this position. The baseline (control) stimuli for the odd-ball paradigm show only an initial onset response. Baseline stimuli consisted of the default stimulus set without inclusion of the deviant or omitted stimulus, while the shifted baseline stimuli consisted of the same stimulus set at the baseline but shifted forwards by one frame to match the timing without incurring a violation.
**(C)** Example unit activations for deviant (i) and omission (ii) stimuli using a less conservative set of criteria. Here, these units show mixed-selectivity and are responsive to both standard and deviant stimuli.

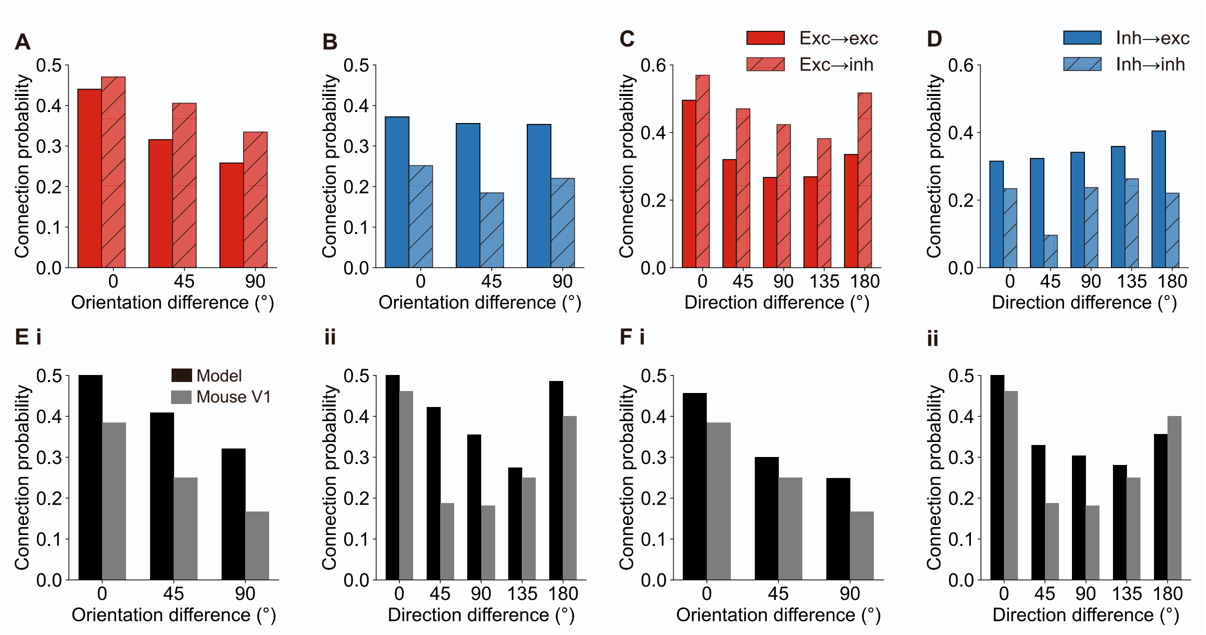


**Figure S3: Orientation and direction dependence of connection probabilities across model unit types, Related to Figure 2.**

**(A, B)** Connection probability as a function of orientation tuning difference for excitatory (A) and inhibitory (B) model units. Excitatory units show a monotonic trend of decreasing connection probability, whereas inhibitory units show a much weaker effect, implying a broader functional spread of inhibitory connections.

**(C, D)** Connection probability as a function of direction tuning difference for excitatory (C) and inhibitory (D) model units. Excitatory units show a characteristic u-shaped curve, as found in V1, where units with similar or opposite direction tuning are most likely to connect. Inhibitory units synapsing with excitatory units show a weak but monotonically increasing trend, where the closer they are to having opposite direction preferences, the more likely they are to connect. Finally, inhibitory model units synapsing with other inhibitory units show a more heterogenous pattern, with no overall preference for a given difference in direction tuning.

**(E, F)**. Connectivity motifs in the model across simple-cell-like (E) and complex-cell-like (F) populations in the model for short-range orientation-dependent connectivity (i), and short-range direction-dependent connectivity (ii).

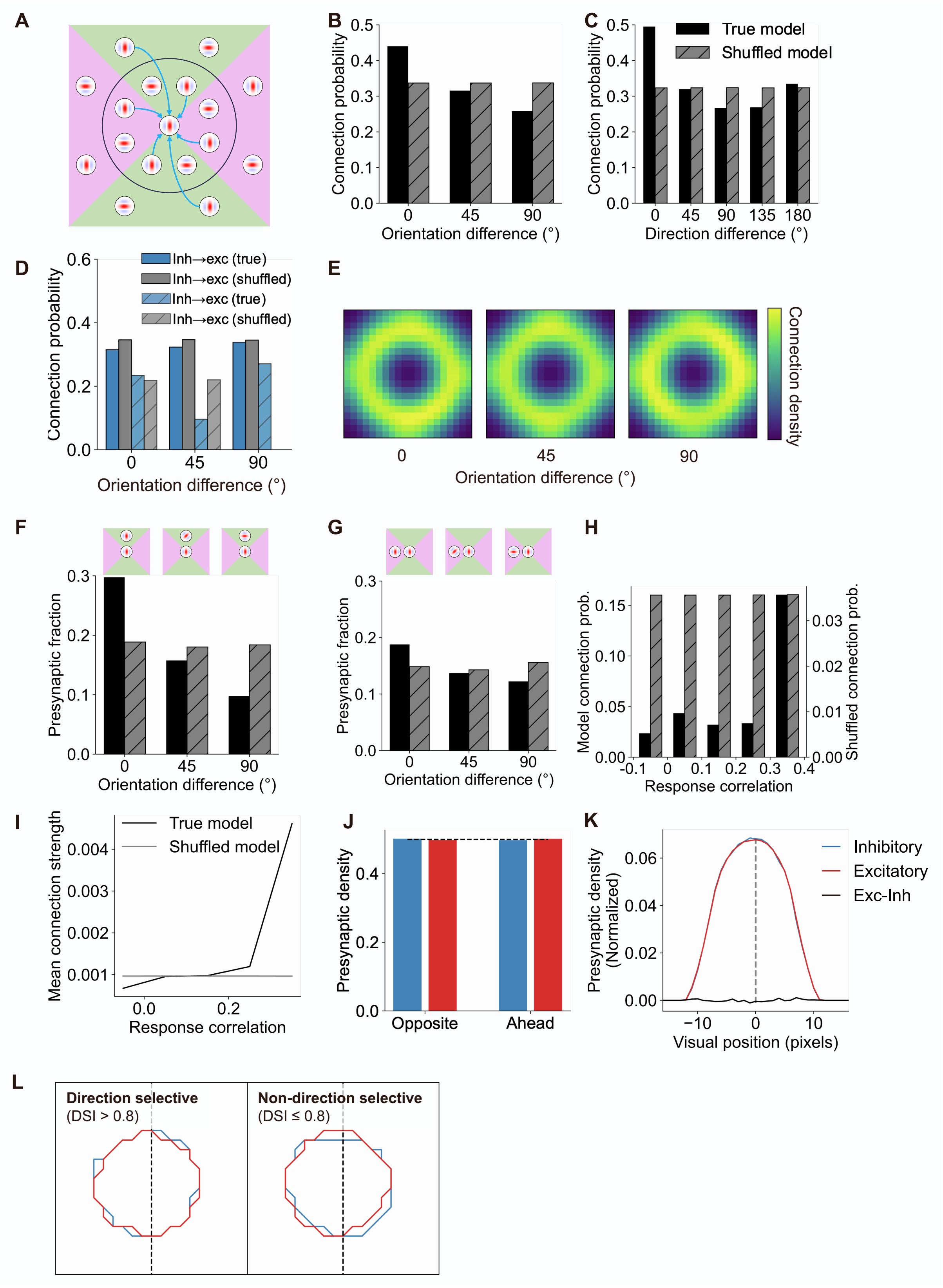


**Figure S4: Functional connectivity resembling mouse V1 in the model is abolished when connectivity is measured with the recurrent weights randomly shuffled, Related to Figures 2 and 3.**

**(A)** Schematic of local functional connectivity in mouse V1.

**(B, C)** Short-range connection probability as a function of the difference in orientation and direction tuning among model units.
**(D)** As in B, but for inhibitory-to-inhibitory and inhibitory-to-excitatory connections in the model.
**(E-G)** Long-range connection probability as a function of difference in orientation preferences for receptive fields located in co-axial (F) and co-orthogonal (G) locations relative to the receptive field of the post-synaptic unit. Heatmap (E) shows the shuffled connection probability over visual space across differences in orientation tuning for model units. Heatmap is smoothed for display purposes with a Gaussian filter ($\sigma$=2 pixels).
**(H, I)** Response correlation for model units as a function of connection probability (H) as well as the input connection strength (I).
**(J)** After shuffling, model unit presynaptic density for excitatory and inhibitory cells is equal in both halves of visual space. Dashed line represents equal density (0.5).
**(K)** Profile of model unit presynaptic density across horizontal visual space for excitatory and inhibitory inputs. Profiles smoothed with a 5-pixel moving average.
**(L)** Pooled density contours across all excitatory (red) and inhibitory (blue) model units for direction- and non-direction-selective post-synaptic excitatory units, showing no overall differences between them after shuffling.

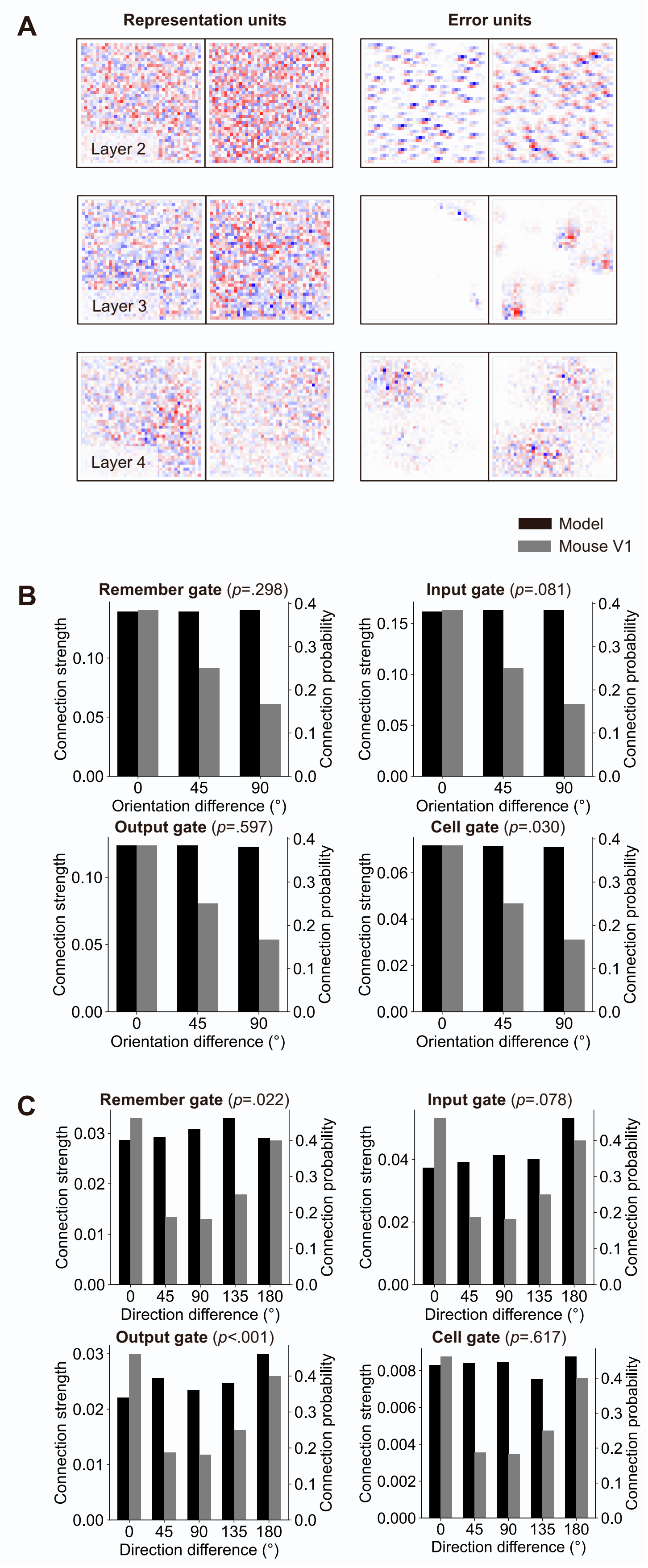

A
Representation units
Error units
Layer 2
Layer 3
Layer 4
Model
Mouse V1
B
Remember gate (p=.298)
Input gate (p=.081)
Output gate (p=.597)
Cell gate (p=.030)
Connection strength
Connection probability
Orientation difference (°)
C
Remember gate (p=.022)
Input gate (p=.078)
Output gate (p<.001)
Cell gate (p=.617)
Direction difference (°)

**Figure S5: PredNet does not recapitulate short-range orientation- or direction-dependent connectivity, Related to Figure 4.**
**(A)** Optimal stimuli for exemplar units in PredNet estimated using gradient ascent. While there is clear structure present in the error units, this structure and any spatial localization are markedly absent in the recurrently-connected representation units.
**(B)** PredNet does not capture well the finding that units with similar orientation tuning are more likely to connect, with little variation in connection strength as a function of orientation difference.
**(C)** PredNet shows more variation in connection strength as a function of the difference in preferred stimulus direction across its recurrent weights, but these properties do not vary in the same way as in V1, with the characteristic ‘U’-shape absent.

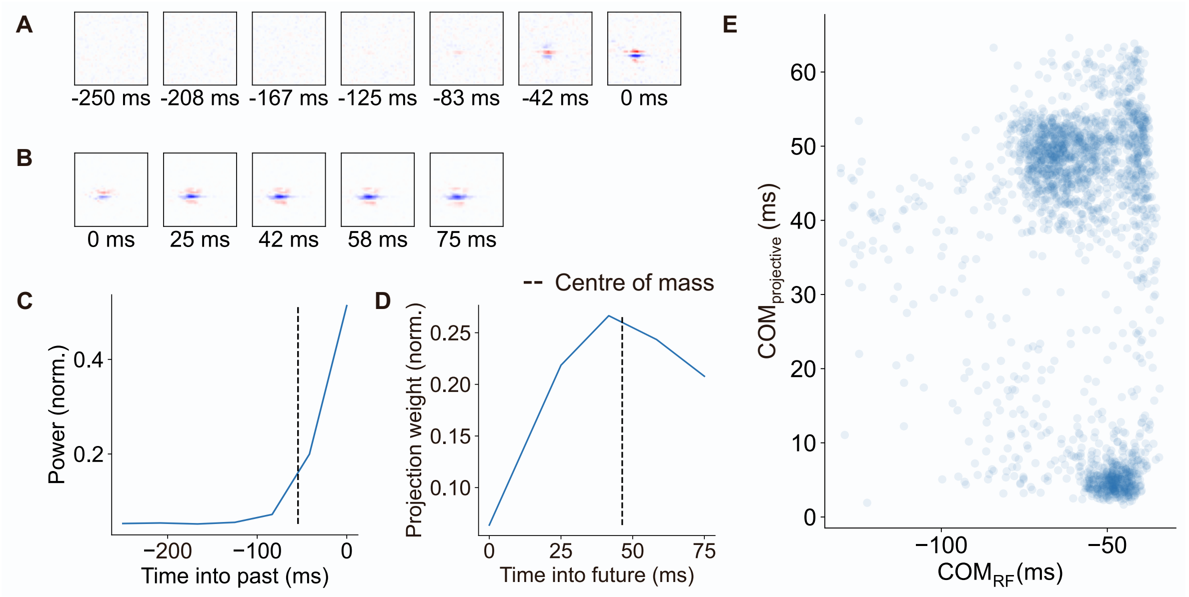


**Figure S6: Units that integrate information further into the past project further into the future in the span-predicting temporal prediction model, Related to Figure 5.**
**(A)** Example spatiotemporal receptive field for a single model unit showing a decay in power into the past.
**(B)** The ‘projective’ receptive field for the same model unit in A, showing this unit’s linear output weights at each future prediction time step.
**(C, D)** Temporal power of the receptive field into the past (C) and of the projection weights into the future (D) for the example shown in A-B. The dashed line indicates the center of mass for each curve.
**(E)** Scatter plot for the center of mass of the temporal power of the receptive fields ($COM_{RF}$) versus the center of mass of the temporal power of the corresponding projective fields ($COM_{projection}$). Each point is a hidden unit.
